# Trait anger modulates neural activity in the fronto-parietal attention network

**DOI:** 10.1371/journal.pone.0194444

**Published:** 2018-04-19

**Authors:** Nelly Alia-Klein, Rebecca N. Preston-Campbell, Scott J. Moeller, Muhammad A. Parvaz, Keren Bachi, Gabriela Gan, Anna Zilverstand, Anna B. Konova, Rita Z. Goldstein

**Affiliations:** 1 Departments of Psychiatry and Neuroscience, Icahn School of Medicine at Mount Sinai, New York, New York, United States of America; 2 Battelle Public Health Research & Translational Science, St. Louis, Missouri, United States of America; 3 Department of Psychiatry, Stony Brook University School of Medicine, Stony Brook, New York, United States of America; 4 Department of Psychiatry and Psychotherapy, Central Institute of Mental Health, Medical Faculty Manheim, Heidelberg University, Heidelberg, Germany; 5 Center for Neural Science, New York University, New York, New York; University of Bologna, ITALY

## Abstract

Anger is considered a unique high-arousal and approach-related negative emotion. The influence of individual differences in trait anger on the processing of visual stimuli is relevant to questions about emotional processing and remains to be explored. Using functional magnetic resonance imaging (fMRI), we explored the neural responses to standardized images, selected based on valence and arousal ratings in a group of men with high trait anger compared to those with normative to low anger scores (controls). Results show increased activation in the left-lateralized ventral fronto-parietal attention network to unpleasant images by individuals with high trait anger. There was also a group by arousal interaction in the left thalamus/pulvinar such that individuals with high trait anger had increased pulvinar activation to the high-arousal (versus low arousal) unpleasant images as compared to controls. Thus, individual differences in trait anger in men are associated with brain regions subserving executive attentional and sensory integration during the processing of unpleasant emotional stimuli, particularly to high arousal images.

## Introduction

Anger is considered to be an approach-related emotional state incorporating physiological, affective, cognitive, and behavioral components which occur in response to unpleasant or undesired events [1–4]. The experience and expression of anger is demonstrated as high-arousal emotional reactivity to negatively valenced stimuli [2–8]. Some individuals are prone to experiencing anger, reflecting an enduring trait pattern of response [6, 9, 10]. Evidence has shown that individuals with elevated levels of anger demonstrate reduced self-control [11] while attributing disproportionate salience and preferentially attending particularly to negatively valenced stimuli or threatening cues [9, 12–14].

Functional neuroimaging studies have implicated a network of brain regions subserving the processing of emotional stimuli encompassing the amygdala, insula, and prefrontal cortical (PFC) areas [e.g., medial and lateral PFC, and the orbitofrontal cortex (OFC)] [15–20]. Further, emotional processing of visual stimuli involves the visuospatial-attentional processing network, including the visual cortex, parietal cortex [21], dorsolateral prefrontal cortex (DLPFC), and thalamus [22]. These networks are also involved in motivated attention to the significance, or sensory distinctiveness of the stimuli, and are modulated by both stimulus valence (i.e., pleasant or unpleasant) and arousal levels [23, 24]. The neural processing of arousal, or the intensity of the experience of an emotion, which can range from calming to exciting or agitating [25], has been associated with activation in the amygdala, insula, ventrolateral PFC, and dorsomedial PFC [25–28].

Currently, the relationship between elevated trait anger and neural activity during emotion processing is not well understood. It is postulated that the approach motivation in anger, modulates the evaluative response to salient stimuli [1, 2, 29]. This motivational theoretical model suggests that cortical regions are asymmetrically involved in approach and avoidance motivation, and that approach-related anger [30], associated with increased levels of left lateralized brain activity [31, 32] and decreased levels of right frontal activity [2] reflects a bias or selective attention to negatively valenced stimuli. These network functions may share characteristic patterns in externalizing psychopathology. For example, individuals with intermittent explosive disorder (IED) and cocaine addiction show hyper-reactivity to error commission and left DLPFC positivity correlated with increased trait anger expression [33]. Others vulnerable to externalizing psychopathology have demonstrated reduced prefrontal cortex [medial (MPC), anterior and posterior cingulate cortex (ACC, PCC) and OFC] and increased insula and subcortical responses (amygdala, hippocampus, thalamus [34–38] on behavioral inhibitory control and emotional tasks; yet, little is known about the neural mechanisms underlying elevated trait anger.

One important element that may modulate the neural response by individuals with high trait anger is autonomic response characterized by overall low level of physiological arousal. We recently documented reduced blood pressure and reduced OFC response to violent video content in a reactive aggressive sample [11]. Cardiovascular hypoarousal is directly associated with neural activity within areas of the anterior cingulate cortex, OFC, medial prefrontal cortices, and the amygdala and often in interaction with activity in the insula, and relay regions of the thalamus and brainstem [22]

In this study we chose to explore the neuronal correlates of viewing images that are normatively considered unpleasant (i.e., of negative valence) in individuals with high trait anger in order to uncover the potential effects of increased attention or sensitivity to negatively valenced cues in this population. Our intent was to determine if individuals with elevated trait anger as compared to controls would demonstrate increased blood oxygenation level dependent (BOLD) activity in left-lateralized brain attention networks that are involved in processing of normatively considered ‘high arousal’ emotional visual stimuli. During fMRI, individuals with elevated trait anger and those with normative trait anger viewed affectively unpleasant and pleasant pictures, which varied on normative population values of subjective arousal (high vs. low). We hypothesized that, relative to controls, men with high trait anger (HTAs) would show left lateralized hyper-reactivity of networks involved in emotional processing and attention allocation, specifically in response to viewing high arousal unpleasant visual stimuli.

Because anger is often a precursor for violent behavior, there are clinically relevant implications to understanding psychiatric conditions that are marked by elevated levels of anger [i.e., IED, antisocial personality disorder (ASPD)]. As such, modulating neural reactivity to aversive stimuli by individuals with high trait anger through biofeedback for example may be a candidate approach for therapeutic intervention.

## Methods

### Ethics statement

This research was approved by the Institutional Review Board (IRB) at Stony Brook University. All individuals provided written informed consent in accordance to the IRB prior to study participation.

### Participants

Healthy individuals and those who felt their anger experiences were problematic were recruited from the general population, through newspaper advertisements and word-of-mouth. Inclusion criteria were for native English speaking and not currently taking any medication. Exclusion criteria were the following: 1) any neurological condition, history of seizures, and/or head trauma with loss of consciousness (>30 minutes); 2) use of any psychoactive medication within 6-months prior to the study; 3) history of cardiovascular (e.g., high blood pressure), endocrinological, metabolic, oncological, or autoimmune diseases; 4) contraindications to MRI; 5) history of major psychiatric disorder other than IED or ASPD; 6) current alcohol intoxication or positive urine screens for psychoactive drugs or their metabolites (amphetamine or methamphetamine, cocaine, phencyclidine, benzodiazepines, cannabis, opiates, barbiturates, or inhalants).

Thirty-seven male participants were grouped via median split using the State-Trait Anger Expression Inventory (STAXI-2) [3, 39, 40]. Specifically, the Trait Anger subscale divided subjects to the Trait Anger (HTAs) group [(N = 20, mean ± standard deviation, 25.5 ± 1.8) and control group (N = 17, 11.8 ± 0.49)]. Using a one-sample t-test, the HTAs’ mean score was significantly higher, and the controls’ was significantly lower, than the normative Trait Anger score at the 50^th^ percentile for men [40] (both *p* < 0.001), indicating that this split was a valid way of partitioning the groups. Participants underwent a comprehensive clinical interview consisting of the Structured Clinical Interview for DSM-IV Axis-I Disorders [41]; the Structured Clinical Interview for Axis-II personality disorders, specifically Cluster B (Antisocial Personality Disorder; ASPD) [42]; and an assessment for IED according to DSM-IV criteria (IED-IR) [43]. The psychiatric disorder IED is defined as the inability to resist aggressive impulses that result in repeated acts of verbal and/or physical aggression that are grossly out of proportion to the experienced provocation, affecting 7% of the US population (lifetime prevalence) [44, 45]. This disorder has been associated with affect dysregulation, clinically substantial features of increased levels of trait anger and deficits in social-emotional information processing [46, 47].

Based on this clinical interview, nine HTAs had a diagnosis related to anger/aggression, of which five met criteria for IED, and four met criteria for ASPD. No psychopathology was found in controls. Importantly, the groups were matched on age, race, handedness, education, and estimates of verbal and non-verbal intelligence (Table 1).

**Table 1:** Demographics and estimates of intelligence for all study participants.

Participants (all male)	Test	*p*-value	HTAs (n = 20)	Controls (n = 17)
^a^Age (years)	t_35_ = 0.87	0.38	34.9 ± 8.3	32.7 ± 6.5
^b^Race (Black/Hispanic/Caucasian/Other)	χ^2^ = 0.95	0.82	12 / 4 / 3 / 1	10 / 4 / 3 / 0
Handedness (right/left)	χ^2^ = 0.01	0.91	19/1	16/1
Education (years)	t_35_ = 0.50	0.62	13.1 ± 1.5	13.3 ± 1.59
Verbal IQ: Wide Range Achievement Test III: Reading Scale	t_35_ = 1.36	0.18	10.6 ± 3.8	12.1 ± 2.3
Non-verbal IQ: WASI—Matrix Reasoning Subtest	t_35_ = 0.98	0.33	10.0 ± 2.7	10.9 ± 2.8

^*a*^ Values are frequencies or means ± standard deviation.

^b^ Race: Other (Asian / more than one race).

### Task

Participants passively viewed a series of IAPS images [48], a normed image bank widely used in studies investigating the neural correlates of emotional processing [26]. This bank is comprised of emotion laden images selected with respect to ratings on the dimensions of valence and arousal. Using normative image values from male raters, and defining levels of arousal based on one standard deviation above or below the mean, we selected an image subset of 100 images (25 per condition) for use in the construction of each unique block presented in the fMRI: unpleasant high-arousal (violence, bodily mutilation, and threat), unpleasant low-arousal (people in distress, accidents), pleasant high-arousal (nudity, erotica), and pleasant low-arousal (nature scenes, infants). Normative means and standard deviations for each image condition and tests comparing the conditions are presented in Table 2.

**Table 2:** Means and standard deviations for valence and arousal for the IPAS pictures in each condition and t-tests testing for significant differences between conditions.

	Mean ^a^Valance	SD	Mean ^a^Arousal	SD
Unpleasant-NA	2.88	.19559	4.58	.22630
Unpleasant-HA	2.7516	.35293	6.0491	.18666
Pleasant-NA	7.0133	.24203	4.4130	.20911
Pleasant-HA	7.2879	.27704	6.6293	.26090
Condition comparison	t-valence	p-value	t-arousal	p-value
Unpleasant-NA vs Unpleasant-HA	1.145	.258	-6.662	< 0.001
Pleasant-HA vs. Pleasant-NA	-1.436	.157	-8.125	< 0.001

^a^ Norm values are taken from Lang PB, MM; Cuthbert BN. International Affective Picture System (IAPS): Affective Ratings of Pictures and Instruction Manual. 2005.

Each image was presented for 6.75 seconds in blocks of four images of the same type (e.g., unpleasant high-arousal) presented serially. Blocks were separated by a fixation cross (inter trial interval) of 20 seconds, presented before the first image block, but not after the last image block. This fixation cross served as the implicit baseline for our fMRI analyses. Participants completed two runs, each with eight unique blocks (32 images per block; total of 64 different images). Both the blocks and the presentation of the images within each block were pseudorandomized and counterbalanced within and across runs, such that blocks of the same condition were not repeated serially and block sequences of four were not repeated in a run (e.g., ABCD, CDBA). During the task, participants viewed the images through MR-compatible goggles; presentation of stimuli was controlled using an IBM-compatible computer running the E-Prime 2.0 software (Psychology Software Tools, Pittsburgh, PA). Participants were instructed to keep their eyes open, not to move their head or body during the scan, and to press a button to confirm viewing each image. Prior to the first experimental run, participants were familiarized with the paradigm by completing a practice run of three blocks of neutral pictures.

After exiting the scanner, a subset of participants (HTAs, n = 12; controls, n = 12) completed ratings for a selection of the fMRI task images (12 images); nine unique sequences of 12 images were created. Each picture was accompanied by two questions, measuring arousal and valence, respectively: Arousal: “How *excited* or *calm* does this make you feel?” Valence: “How *pleasant* or *unpleasant* does this make you feel?” All responses were recorded using a visual analogue scale (1–9; where 1 designated no arousal or unpleasant and 9 designated high arousal or pleasant).

### Functional MRI

Functional magnetic resonance imaging was performed on a 4T whole-body Varian/Siemens MRI scanner. The BOLD-fMRI responses were measured as a function of time using a T2*-weighted single-shot gradient-echo planar sequence (TE/TR = 20/1600 ms, 4 mm slice thickness, 1 mm gap, 33 coronal slices, 20 cm FOV, 64 × 64 matrix size, 90°-flip angle, 200 kHz bandwidth with ramp sampling, 470 time points, and 4 dummy scans to avoid non-equilibrium effects in the fMRI signal). Earplugs (28 dB sound attenuation; Aearo Ear TaperFit 2; Aearo Company) and headphones (30 dB sound attenuation; Commander XG MRI Audio System, Resonance Technology Inc.) were used to minimize scanner noise [49].

### Image preprocessing and statistical analyses

Data were pre-processed and analysed using SPM8 (Wellcome Department of Cognitive Neurology, London UK) (http://www.fil.ion.ucl.ac.uk/spm) running on MATLAB 2007b (Mathworks Inc., Natick, MA). A six-parameter rigid-body transformation (3 rotations, 3 translations) was used for image realignment and to correct for head motion. Criteria for acceptable motion were <2 mm displacement and <2° rotation in any axis in any task run. The realigned datasets were spatially normalized to the standard stereotactic space of the Montreal Neurological Institute (MNI) using a 12-parameter affine transformation (3 translations, 3 rotations, 3 shears, 3 zooms), and a voxel size of 3-mm^3^. An 8-mm full-width-half-maximum Gaussian kernel spatially smoothed the data.

For first-level analysis, images were thresholded using the default masking threshold of 0.8. To calculate individual BOLD-fMRI maps for the task, which has a blocked design comprising 470 time points, a general linear model and a box-car design convolved with a canonical hemodynamic response function and high-pass filter (cutoff frequency 1/800s) was used. Four contrast images per participant were calculated for each of the image conditions (unpleasant low-arousal, unpleasant high-arousal, pleasant low-arousal, pleasant high-arousal). The second-level analysis was conducted to determine of the effects that are observed in the single-subject level differ as a function of group.

On the second—level, between-group differences and potential interactions were assessed with two separate valance-based flexible factorial models were estimated in SPM8 with a within-subjects factor of arousal (high, low) and a between-subjects factor of group (HTAs, control) using the contrast images mentioned above. Specifically, in the first design, we modeled the effects of brain response to arousing pictures during viewing of unpleasant images using a 2 (image type: unpleasant high-arousal, unpleasant low-arousal) × 2 (group: HTAs, controls) mixed design, and in the second, the effects of brain response to arousing pictures during viewing of pleasant images using a 2 (image type: pleasant high-arousal, pleasant low-arousal) × 2 (group: HTAs, controls) mixed design. Note that we did not perform a 2x2x2 (valance x arousal x group) because we did not have sufficient power to explore a 3-way interaction. To test for significance, a voxel-wise threshold of *p* < 0.005 was applied, combined with a minimum cluster-extent of 26 contiguous voxels (702 mm^3^), to yield a corrected cluster-level false positive rate of *p*<0.05 as determined by Monte Carlo simulations (similar to AlphaSim) [50] (http://www2.bc.edu/~slotnics/scripts.htm). We examined any interaction effects statistically in SPSS using the extracted cluster values from Marsbar (http://marsbar.sourceforge.net/index.html) in order to determine, through post-hoc comparisons, what BOLD response was driving the interaction.

**Fig 1:**
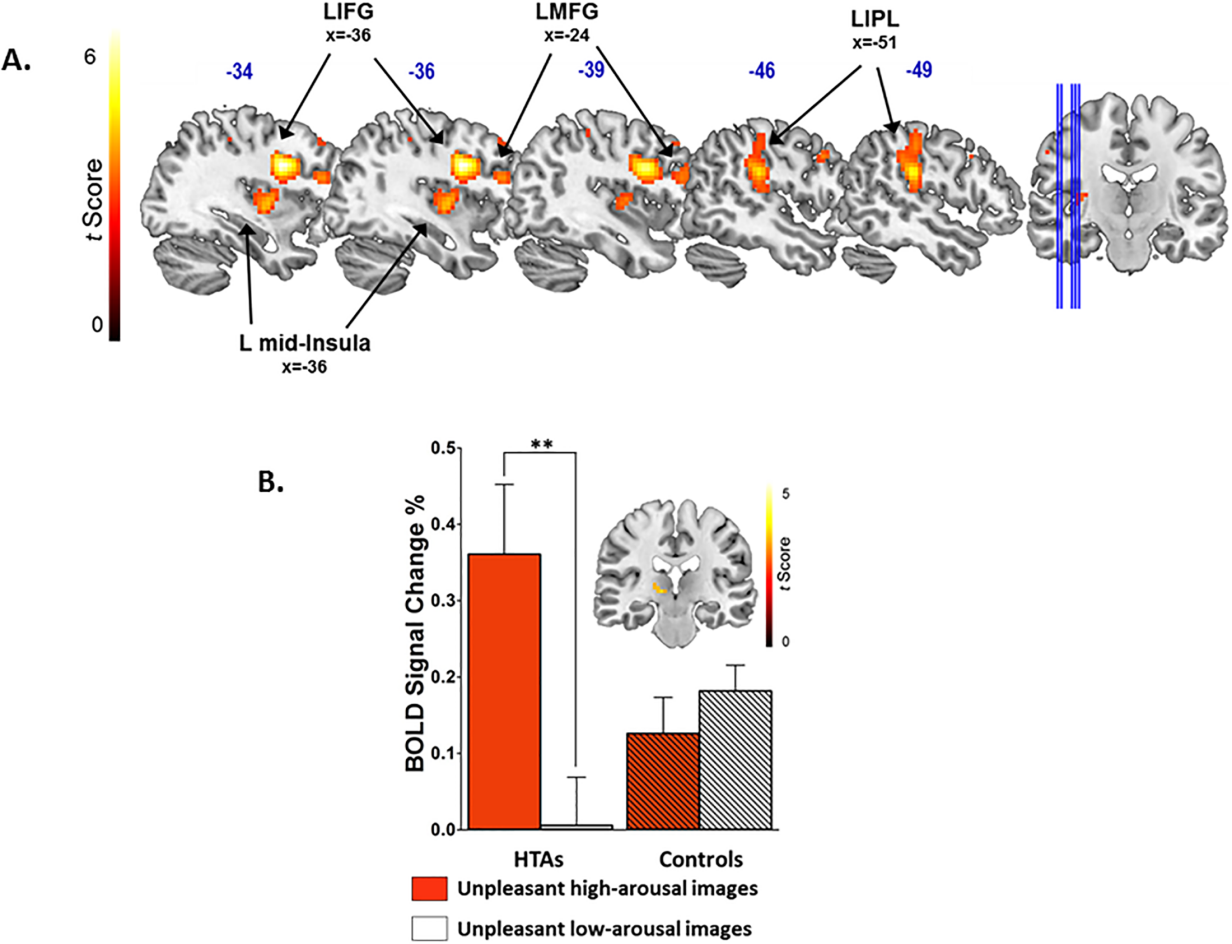
Brain response to unpleasant images. **(A)** Main effect of group (HTAs > controls) for unpleasant images across arousal conditions. The HTAs exhibited increased activations within the left IFG/precentral gyrus, MFG, insula, and IPL compared with the control group when viewing unpleasant images. **(B)** A significant group × arousal interaction for unpleasant images emerged in the left thalamus (pulvinar) driven by the difference in brain response for arousal (high>low) within the HTAs [open bars; t(19) = 3.20, *p* = 0.003]. In the control group, no difference in the brain response to unpleasant images [diagonal filled bars; t(16) = -0.62, *p* = 0.54] emerged. Whole-brain significance threshold was set to *p* < 0.005, combined with a minimum cluster-extent of 26 contiguous voxels (702 mm^3^), to yield a corrected cluster-level false positive rate of *p* < 0.05. IFG = inferior frontal gyrus, MFG = middle frontal gyrus, IPL = inferior parietal lobule. Red bars indicate unpleasant high-arousal images; white bars indicate unpleasant low-arousal images. ** p < 0.01.

The average percent signal change for all significant clusters were extracted with and were used to inspect for outliers (i.e., three standard deviations from the mean). No extracted fMRI-BOLD signals for any significant cluster were outside this range; therefore, no data were discarded from the analysis. Extracted data values were used to present the data in graphical form. T-maps were used to present significant clusters (Fig 1B).

### Image ratings

To compare the subjective effects of arousal and group for the unpleasant images, we conducted a 2 x 2 repeated measures analysis of variance (ANOVA) with picture arousal (low vs. high) as the within subjects variable, and group (HTA vs. controls) as the between subjects variable. There was indeed a significant main effect for arousal [unpleasant: F(1,22) = 12.51, *p* = 0.002], validating the high and low-arousal distinction (high > low) in the task. Neither a main effect of group [unpleasant: F(1,22) = 2.76, *p* = 0.11], nor a group × arousal interaction [unpleasant: F(1,22) = 2.07, *p* = 0.16] reached significance [26]. Identical analyses were used to compare subjective effects of arousal for pleasant images (See Supporting Information).

Finally, since nine participants within HTAs had anger and/or aggression diagnosis (i.e., IED or ASPD) we conducted additional analyses on brain response to the IAPS images comparing these diagnosed participants to those without a diagnosis related to anger/aggression within HTAs. Within the HTA group only, using SPSS, we compared the brain response between participants with an anger/aggression diagnosis (i.e., IED or ASPD, n = 9) to those participants in the HTA group that did not meet criteria for and anger/aggression diagnosis (n = 11). Here we used independent t-test to compare the extracted values obtained in MARSBAR) for all significant clusters between the groups. No group differences were noted in brain response, or in demographic variables between participants (p > .26).

## Results

### Brain response to unpleasant images

There was a main effect of group for unpleasant images across arousal conditions such that HTAs showed higher left lateralized activations in the middle occipital gyrus (MGO), inferior frontal gyrus (IFG), middle frontal gyrus (MFG), mid-insula, and inferior parietal lobule (IPL) compared with controls (Fig 1A). Importantly, a group × arousal interaction emerged in the left pulvinar/thalamus. Post-hoc analyses revealed that, as compared to controls, HTAs had increased responses for high > low arousal unpleasant images (Fig 1B). Table 3 provides these and additional results found during the whole-brain analyses of brain response to unpleasant images. Further analyses showed that diagnosis of IED and ASPD did not drive these results within HTAs. Imaging results for pleasant images are found in the supporting information, S1 Table.

**Table 3:** Significant activations to unpleasant images revealed by whole brain analysis.

Contrast	aBrain Region	X	Y	Z	T	Cluster Size
**Main Effect of Arousal**						
**High > Low**	none					
**Low > High**						
	Left ACC (BA 32)	-15	38	16	3.75	54
	Right ACC (BA 32)	15	47	19	2.96	37
**Main Effect of Group**						
**High Trait Anger > Control**						
	Left MOG (BA 18)	-27	-97	4	3.69	44
	Left IFG (BA 6)	-36	2	31	5.81	163
	Left MFG (BA 46)	-42	35	28	3.35	73
	Left Insula (BA 48)	-36	-7	4	3.75	62
	Left IPL Supramarginal (BA 40, 3)	-51	-28	25	4.44	210
**Control > High Trait Anger**						
	Right Precuneus (BA 19)	27	-82	34	4.71	73
	Right Red Nucleus	0	-13	-8	4.69	260
	Right Precuneus (BA 7)	21	-58	58	3.97	62
	Right Postcentral (BA 5)	18	-55	70	3.97	54
	Right Lingual (BA18)	12	-70	4	3.53	32
**Group × Arousal Interaction**						
	Left Thalamus/pulvinar	-12	-22	1	3.01	30

aFor whole brain analysis, a voxel-wise threshold of P < 0.005 was applied, combined with a minimum cluster-extent of 26 contiguous voxels (702 mm^3^), to yield a corrected cluster-level false positive rate of *p* < 0.05. This table includes the coordinates x, y, and z of the peak voxel given in Montreal Neurological Institute space and their statistical significance (t-values). BA, Brodmann area. ACC, anterior cingulate cortex; MOG, middle occipital gyrus; IFG, inferior frontal gyrus; MFG, middle frontal gyrus; IPL, inferior parietal lobule.

## Discussion

In the current study, we investigated the influence of trait anger on the neural response to unpleasant and pleasant visual stimuli, selected based on high and low arousal. To the best of our knowledge, this is the first fMRI investigation that identifies distinct neural circuitry associated with the processing of unpleasant images as a function of elevated trait anger. We documented unique left lateralized activation in the IFG, MFG, and mid-posterior insula during viewing of unpleasant images in HTAs as compared to controls. We further found that HTAs had increased BOLD signal to high-arousing but not low-arousing unpleasant images relative to controls in the thalamus/pulvinar.

More specifically regarding the first result, we found increased engagement of the salience and the fronto-parietal attention networks (i.e., MFG/IFG, mid insula, and parietal regions) in HTAs relative to controls in response to viewing the unpleasant pictures [24]. The PFC and insula are part of a salience network that has extensive connectivity with the subcortical structures that underlie interoceptive autonomic processing [23]. Activation in this network, strongly influenced by motivational salience of the stimuli, was heightened while viewing negative images [51]. Prefrontal regions and insula are involved in the appraisal of emotional stimuli [52–54], emotion regulation, expression of emotion, explicit threat evaluation [55], and salience detection [23, 24]. The insula is commonly activated in tasks that are associated with the processing of negative emotion reactivity [56, 57] such as disgust, sadness [58–60], negative and visceral affective sensation and integration [53, 58, 60]. Although part of the salience network, the insula has also been found to coactivate with the ventral fronto-parietal attention network, with greater insular activity reflecting increased attentional bias to salient stimuli [61]. It has been postulated that increased activation of this region of the insula in emotion tasks involves initiation of attentional control, while engaging higher-order control processes [62] in tandem with salience discrimination and integration of sensory information [63]. Taken together, this pattern of neural activity may indicate emotional arousal through activation of the attentional and salience networks.

Increased activation levels in IFG and mid-posterior insula in HTAs was also associated with increased inferior parietal lobule activity, supporting an increased recruitment of the ventral fronto-parietal attention network in individuals with high anger [24, 64]. Within this network, the IFG, as part of the broader DLPFC, has been implicated in cognitive control and plays an important role in the processing of emotion mechanisms[65]. Activation of this region has been observed during various emotional processes such as emotion recognition and evaluation [66, 67] and emotional perspective taking [68] with the degree of activation correlating with the degree of interpersonal involvement with the stimuli [68]. In this task, activation in the DLPFC potentially reflects an activation of the frontoparietal attention network, suggesting increased attention to the unpleasant images. Thus, one interpretation of the increased activation in the IFG in the HTA group in response to the unpleasant pictures is that the effect of increased activation relative to controls was due to a greater attentional bias towards threatening pictures in men who exhibit elevated levels of trait anger. This interpretation, however, must be tested more directly in future studies. Given the relatively modest effect sizes in these results it is probable that stronger effects can be attained through experimental paradigms that have a clear component of attentional effort.

There was no finding of self-report arousal differences between the groups. However, perhaps supporting a heightened arousal specific to HTAs in response to unpleasant images, we found a group × arousal interaction in the left pulvinar. The pulvinar has an integrative function in bottom-up visual attention allocation functional loops, linking it to selective attention [69–74] to motivationally relevant features (salience) of unpleasant visual stimuli [75]. Additionally, it plays an important role in coordinating and refining affective processing in dorsal and ventral (what and where) visual cortices involved in visual processing [76, 77]. Here it appears that unpleasant high-arousal images differentially activate the pulvinar, thereby highlighting its role in automatic, directed attention and the signaling of the salience of the visual stimuli. It is important to note that a valid measure of attentional bias is needed to show a correlation with pulvinar signals in future studies to further validate our hypothesis. Likewise, a behavioral response directly measuring anger after each trial could increase the observed effects and would be better linked the fMRI findings.

Across conditions, consistent with our hypotheses, group differences observed in response to unpleasant images appeared lateralized, where HTAs showed activations on the left hemisphere and controls showed activations on the right hemisphere. This lateralization effect is in line with a motivational system theory that relate aspects of the experience and expression of emotions such as anger, sadness, and fear [31, 32, 78] to specific patterns of regional brain activity. Here, an approach system, involved in approaching/attending to rewarding or appetitive stimuli is associated with greater left than right frontal activity, and an avoidance system, associated withdrawal/avoidance from aversive stimuli, is associated with greater right than left frontal activity [1, 32, 79, 80]. Evidence suggests that individuals high in trait anger [30] exhibit increased levels of assertiveness and competitiveness which is a subcomponent of novelty seeking, that is itself associated with approach motivation [32] and left-dominant frontal neural activity [31]. Taken together, the increased left lateralized brain responses within the ventral fronto-parietal attention network suggest that individuals high in trait anger may have an attentional bias towards the unpleasant images which might be mediated by approach motivation. Further, right cortical activation seen in the control group may be involved in the modulation of arousal and threat response and avoidance-oriented attentional processes. These differences in activation patterns between groups may stem specifically from basic constitutional differences that underlie trait processes, since within HTAs those with psychiatric diagnosis did not differ from those without a diagnosis.

The present study focused on males. The question whether our results extend to female adults with high trait anger is a direction for future research. Although the amygdala is the structure most implicated in emotional processing [81, 82] we did not find amygdala activation in either HTAs or controls to the visual stimuli, and more specifically the unpleasant images. One potential reason for lack of amygdala activation could be the configuration of the stimuli presented in one block (pleasant or unpleasant—varied on arousal) may influence the participant’s processing for subsequent stimuli. Moreover, activation of the amygdala might not have been detected by the block design of the fMRI paradigm given that the amygdala habituates rapidly under aversive or fear stimulation [83, 84]. Additionally we examined the processing of emotional visual stimuli in a high trait anger group which included participants with heterogeneous disorders characterized by aggressive symptoms (ASPD and IED). In the future, the use of a larger sample would allow for direct comparison between these subgroups.

In conclusion, individuals high in trait anger demonstrate a unique pattern of neural activity distinguished by increased activation in the ventral fronto-parietal attention—salience networks during passive viewing of unpleasant images. Importantly, the activation in the pulvinar further supports our notion of automatic attentional bias to high-arousal unpleasant images in HTAs. In some cases, this increased activation may be associated with biased visual attention and increased engagement which were not observed in response to other types of highly arousing visual stimuli (e.g. pleasant, or pornographic images; supplement). Thus, this study is an important first step in exploring the neural correlates of emotion processing in angry individuals, a direction of research that is essential for uncovering potential effects of increased attention or sensitivity to negatively valenced cues. Insofar as anger is often a precursor to aggressive behavior, our results could also have important clinical implications and are relevant to understanding psychiatric conditions such as antisocial personality disorder, intermittent explosive disorder, and depression that are marked by increased negative emotions such as anger. Reducing the reactivity to salient aversive stimuli of these specific regions/systems through biofeedback and/or pharmacological interventions may be a candidate approach for novel therapeutics.

## Supporting information

S1 Table Results and discussion. Functional MRI activations to pleasant IAPS conditions of high and low arousal, inclusive of results and discussion on response to pleasant images.
(DOCX)
