# Supplementary material for: Trait anger modulates neural activity in the fronto-parietal attention network
Source: PLoS One. 2018 Apr 19;13(4):e0194444. doi: 10.1371/journal.pone.0194444 (PMC5908080; doi:10.1371/journal.pone.0194444)
Supplement: S1 Table — Functional MRI activations to pleasant IAPS conditions of high and low arousal, inclusive of results and discussion on response to pleasant images. (DOCX) [file pone.0194444.s001.docx]

**Supporting Information**

**Results**

**Subjective Rating Response to Pleasant Images**

To compare the subjective effects of arousal and group for the Pleasant images, we conducted a 2 x 2 repeated measures analysis of variance (ANOVA) with picture arousal (low vs. high) as the within subjects variable, and group [high trait anger (HTA) vs. controls] as the between subjects variable. Rating results revealed a significant main effect for arousal [pleasant: F(1,22) = 14.02, *p* = 0.001], validating the high and low-arousal distinction (high > low) in the task. Neither a main effect of group [pleasant: F(1,22) = 1.29, *p* = 0.27], nor a group × arousal interaction [pleasant: F(1,22) = 3.53, *p* = 0.07] reached significance.

**Brain Response to Pleasant Images**

In brain response there was a main effect of group for pleasant images, such that across arousal conditions, HTAs showed greater activation in the left superior frontal gyrus, and decreased activation in the right precuneus and left lingual gyrus compared with controls. A group × arousal interaction emerged in the precentral gyrus extending into the posterior cingulate cortex. This interaction was driven by a higher response in HTAs relative to controls to in the pleasant high-arousal condition, but no group differences were found in the low-arousal condition. Correlations with the pleasant image condition brain areas exhibiting group differences, and correlations with valence ratings did not reach significance. A diagnosis of intermittent explosive disorder (IED) and antisocial personality disorder (ASPD) did not drive these results. Table 1S lists all results for whole brain analysis on pleasant images.

**S1 Table. Significant activations to pleasant images revealed by whole brain analysis.**

| **Contrast** | **Brain Structure** | **X** | **Y** | **Z** | **T** | **Cluster Size** |
| --- | --- | --- | --- | --- | --- | --- |
| **Pleasant Images: Effect of Arousal Across Group** | |  |  |  |  |  |
| **High-Arousal > Low-Arousal** |  |  |  |  |  |  |
|  |  |  |  |  |  |  |
|  | IFG-R (BA 44) | 45 | 11 | 31 | 4.67 | 82 |
|  | Precentral Gyrus- L (BA 6) | -42 | -1 | 34 | 3.97 | 51 |
|  | Angular Gyrus-R (BA 7) | 27 | -58 | 46 | 3.78 | 118 |
|  | SPL – L (BA 7) | -18 | -61 | 52 | 3.41 | 51 |
|  | Thalamus-R | 3 | -4 | 4 | 3.39 | 48 |
|  | SMA- L (BA 8) | -9 | 23 | 58 | 3.31 | 39 |
|  | Superior Medial Gyrus- L (BA 10) | -9 | 56 | 25 | 3.06 | 32 |
| **Low-Arousal > High-Arousal** |  |  |  |  |  |  |
|  | Mid-Insula-R (BA 48) | 36 | -4 | 7 | 4.28 | 164 |
|  | Rolandic Operculum- L (BA 48) | -48 | -7 | 4 | 3.20 | 52 |
| **Pleasant Images: Main Effect of Group Across Arousal** | |  |  |  |  |  |
| **High Anger > Control** |  |  |  |  |  |  |
|  | Superior Frontal Gyrus – L (BA6) | -27 | -7 | 64 | 3.37 | 33 |
| **Control > High Anger** |  |  |  |  |  |  |
|  | Superior Occipital/Precuneus-R (BA 19) | 24 | -79 | 34 | 3.55 | 36 |
|  | Lingual gyrus- L (BA18) | -12 | -73 | -5 | 3.34 | 28 |
| **Group x Arousal Interaction** |  |  |  |  |  |  |
|  | Precentral gyrus (extends into PCC)-L (BA 6) | -21 | -22 | 52 | 3.44 | 74 |

*a* For whole brain analysis, a voxel-wise threshold of P < 0.005 was applied, combined with a minimum cluster-extent of 26 contiguous voxels (702 mm3), to yield a corrected cluster-level false positive rate of *p* < 0.05. Table includes the activations’ coordinates x ,y, and z of the peak voxel given in Montreal Neurological Institute space and their statistical significance (t-values). BA, Brodmann area; R, right; L, left; IFG, Inferior Frontal Gyrus; SPL, superior parietal lobule; SMA, Supplemental Motor Area; PCC, Posterior cingulate cortex.

**Discussion**

In line with our hypothesis, the overall arousal effects observed across groups is consistent with existing literature involving sexually arousing visual stimuli, thus, we can assume that similar networks are recruited for processing of pleasant high-arousal stimuli in our male participants. Specifically, when viewing pleasant images our data revealed that erotic images induced higher activity than non-erotic images (high > low arousal) in regions associated with sexual arousal such as the parietal areas (superior and inferior parietal lobes), the medial prefrontal cortex, supplemental motor area, and thalamus [1-5]. Activation in parietal areas are associated with the evaluation of complex, emotionally relevant stimuli [2] and it is thought that motivational state and internal drives as they relate to salient visual stimuli mediates the activation of these regions [6]. Involvement of the thalamus has been shown to be associated with the emotional dimension of pleasantness (or valence) of the erotic images [1, 5], while the activation in the left supplemental motor area has been associated with motor imagery in relation to sexual behavior [2].

From the group differences results, it is indicated that HTAs had activation in the left superior frontal gyrus (HTAs > controls) for pleasant images, however these did not correlate with subjective reports. The significant activation in HTAs in the posterior cingulate cortex and superior frontal gyrus, regions involved in attentional networks, to pleasant images suggests that HTAs were recruiting additional attentional resources when viewing pleasant images [2] relative to controls.

References

1. Walter M, Bermpohl F, Mouras H, Schiltz K, Tempelmann C, Rotte M, et al. Distinguishing specific sexual and general emotional effects in fMRI-subcortical and cortical arousal during erotic picture viewing. NeuroImage. 2008;40:1482-94.

2. Mouras H, Stoleru S, Bittoun J, Glutron D, Pelegrini-Issac M, Paradis AL, et al. Brain processing of visual sexual stimuli in healthy men: a functional magnetic resonance imaging study. NeuroImage. 2003;20:855-69.

3. Meseguer V, Romero MJ, Barros-Loscertales A, Belloch V, Bosch-Morell F, Romero J, et al. Mapping the apetitive and aversive systems with emotional pictures using a block-design fMRI procedure. Psicothema. 2007;19:483-8.

4. Arnow BA, Desmond JE, Banner LL, Glover GH, Solomon A, Polan ML, et al. Brain activation and sexual arousal in healthy, heterosexual males. Brain : a journal of neurology. 2002;125:1014-23.

5. Redoute J, Stoleru S, Gregoire MC, Costes N, Cinotti L, Lavenne F, et al. Brain processing of visual sexual stimuli in human males. Human brain mapping. 2000;11:162-77.

6. Critchley HD, Harrison NA. Visceral influences on brain and behavior. Neuron. 2013;77:624-38.
